# Markov-Gibbs Random Field Model for Improved Full-Cardiac Cycle Strain Estimation from Tagged CMR

**DOI:** 10.1186/1532-429X-14-S1-P258

**Published:** 2012-02-01

**Authors:** Matthew J Nitzken, Ayman S El-Baz, Garth M Beache

**Affiliations:** 1 Department of Bioengineering, University of Louisville, Louisville, KY, USA; 2 Department of Radiology, University of Louisville, Louisville, KY, USA

## Background

Due to the ability of MR to index systolic and diastolic non-transmural function, by strain and strain rates, with full 3-D geometric concordance, this area is of ongoing interest for regional ischemic conditions, and for conditions such as heart failure and diabetes that result in global dysfunction. Quantitative tag analysis is often hampered by noisy images, and the tag spatial profile, especially when combined with the very efficient algorithms that are based on frequency or spectral domain analysis. We herein propose a new energy minimization approach, using a 3-variable function (x-direction, y-direction, and time) based on a rotation-invariant, Markov-Gibbs Random Field (MGRF) spatial interaction model. This is intended to reduce noise within a tag line, and un-sharpen the tag edges in the spatial domain (which improves the power spectral density), and thus emphasizes the first symmetric lobes of the Fourier Transform of the images in the frequency domain, for optimized analysis by algorithms such as the Harmonic Phase (HARP) method.

## Methods

The tag-to-background contrast is optimized by modeling the marginal distribution of MR signals for the tag grids and for the anatomic background, by a linear combination of discrete Gaussians (LCDG). The 3-variable MGRF then uses the derived parameters as inputs. This approach requires no user specification, and unique parameters are empirically calculated from the given image. The result is a processed image that is optimized for the frequency domain. We have validated our approach using commercially available HARP analysis software (Diagnosoft Inc.) on synthetic phantoms, and on full cardiac-cycle real data.

## Results

We tested the approach on two synthetic phantoms. We corrupted the phantom images by adding noise. We then calculated the absolute strain error using the HARP technique. In the first phantom the absolute strain error was 94%, and in the second phantom the absolute error was 80%. After processing the images using our approach, the absolute strain error was reduced to 36%, and 16%, respectively. We also observed an average improvement of 230% in the power of the spectral main lobe. We have also generated strain function curves, and parametric strain maps, in patients with ischemic damage from heart attacks who are undergoing experimental myoregeneration therapy, and have documented an improvement in visualization and display of treatment effects, in initial patient data.

## Conclusions

Our results document that Markov-Gibbs Random Field modeling of the spatial domain, for tagged MR images, leads to an improved representation in the spectral domain. This results in more efficient and robust estimation of functional parameters, using spectral domain techniques, such as HARP.

## Funding

None.

